# Using neoadjuvant chemotherapy and replanning intensity-modulated radiotherapy for nasopharyngeal carcinoma with intracranial invasion to protect critical normal tissue

**DOI:** 10.1186/1748-717X-8-226

**Published:** 2013-10-02

**Authors:** Xiaoshuang Niu, Xi Chang, Yunsheng Gao, Chaosu Hu, Lin Kong

**Affiliations:** 1 Department of Radiation Oncology, Shanghai Cancer Center of Fudan University, 270 Dong’an Road, Shanghai 200032, China

**Keywords:** Nasopharyngeal carcinoma, Replanning IMRT, Comprehensive treatment

## Abstract

**Purpose:**

To investigate the feasibility of neoadjuvant chemotherapy and replanning intensity-modulated radiotherapy (IMRT) for intracranial invasion nasopharyngeal carcinoma (NPC).

**Methods and materials:**

From June 2007 to January 2012, 32 patients with intracranial invasion NPC treated with TPF (docetaxel 75 mg/m2, cisplatin 75 mg/m2, 5-FU 2500 mg/m2 every 3 weeks for 3 cycles) neoadjuvant chemotherapy, and replanning IMRT with concurrent chemotherapy were retrospectively studied. The first IMRT plan for each patient was generated based on the original planning CT scan acquired before the start of treatment. Because of tumor shrinkage during radiotherapy, modified gross tumor volume of primary tumor (GTV-P) and high risk clinical target volume (CTV-H), and a new plan was generated and used to complete the course of IMRT. The DVHs of IMRT plan with or without replanning were compared.

**Results:**

There weren’t statistically significant differences in the V95, D-mean, D-95, and D-99 to the modified PTV_GTV-P_ and PTV_CTV-H_ with and without replanning IMRT. Replanning reduced the doses to the brain stem, optic nerve, optic chiasm and temporal lobe. Objective responses were 100.0% 3 months after completion of radiotherapy. Acute toxicities were well tolerated, except for the relatively high incidence of neutropenia. The 2-year local control rates and distant-metastasis free survival were 88.2% (95% CI, 72.9% to 100.0%) and 89.6% (95% CI, 75.9% to 100.0%).

**Conclusion:**

Neoadjuvant chemotherapy and replanning IMRT according to tumor shrinkage during the treatment is essential to ensure safe doses to normal tissues, and produces encouraging outcome for intracranial invasion NPC.

## Introduction

Radiation therapy (RT) is the mainstay of definitive therapy for nonmetastatic nasopharyngeal carcinoma (NPC). New radiotherapy technique, intensity-modulated radiotherapy (IMRT) has allowed improved dose delivery to NPC tumors while reducing dose to normal tissues [1-4]. However, for patients with intracranial invasion NPC, a common problem encounters in planning IMRT is that the vital structures (such as brain stem, optic nerve, chiasm) are in close proximity to the tumor volumes, which creates a dilemma in dose optimization as the region of overlap between the target and the normal tissues [5-10].

NPC is sensitive to chemotherapy. Neoadjuvant chemotherapy has potential advantages of shrinking the tumor bulk before irradiation, as a result narrowing down the tumor target area facilitating the radiotherapy particularly for patients with extensive local infiltration adjacent to critical neurologic structures. The response rate (76.5% ~ 82.0%) of NPC after neoadjuvant chemotherapy with various regimens was reported [11,12]. Furthermore, it was reported [13] the volume of primary tumor declined in 70% ± 4.8% patients after 45 Gy of radiation, which allowed potential target volume and/or dose modification thus improves the therapeutic ratio. Thus, replanning during the course of IMRT may have potential advantages to ensure adequate doses to target volumes and safe doses to normal tissues.

To maximize local control of the tumor and spare the adjacent critical neurologic structures for this group NPC patient with intracranial invasion, from June 2007, we used neoadjuvant chemotherapy and replanning IMRT for 32 patients with intracranial invasion NPC. This study investigates the dosimetric effects of replanning during the course of IMRT on both normal tissues and target volumes. In addition, we report 2-year clinical outcomes of this treatment strategy.

## Materials and methods

### Patient selection

From June 2007 to January 2012, 32 patients with intracranial invasion NPC treated with neoadjuvant chemotherapy and replanning IMRT with concurrent chemotherapy were studied. The disease was staged according to the 2002 American Joint Committee on Cancer (AJCC) staging classifications. Biopsy at the primary site is required for all patients for pathologic diagnosis. Pretreatment staging evaluations included clinical examination of the head and neck, MRI scans of the head and neck, CT scan of the thorax, whole-body bone scan, abdominal sonography, complete blood count, and serum biochemical profile.

### Chemotherapy

All patients were treated with TPF (docetaxel 75 mg/m2, cisplatin 75 mg/m2, 5-FU 2500 mg/m2 every 3 weeks for 3 cycles) neoadjuvant chemotherapy, and replanning IMRT with concurrent chemotherapy. Dose modifications were based on the preceding cycle nadir blood counts and interim toxic effects. A reduction of dosage of docetaxel by 20% with constant cisplatin and 5FU dosages was allowed if a grade IV hematology adverse event or febrile neutropenia emerged in the previous course. All of the dosages of docetaxel, cisplatin, and 5FU were decreased by 20% if more than grade III mucositis or diarrhea happened in the former course.

Concurrent chemotherapy consisted of weekly cisplatin (40 mg/m ^2^) during radiotherapy for a maximum of six cycles, beginning on the first day of radiotherapy as planned. Chemotherapy at the full dose was delivered strictly. The chemotherapy time was postponed if neutropil <2000/lL or platelets < 100,000/lL and suspended if the creatinine clearance rate became < 50 mL/min.

### Radiotherapy and treatment planning

Before treatment, all the patients were immobilized with a thermoplastic head and shoulder mask, and CT simulation according to standard procedures. The CT scan was performed after the completion of neoadjuvant chemotherapy using 0.3 cm slice spacing through the region that contained the primary target volumes, and 0.5 cm through the regions above and below the target volume. Magnetic resonance scans and fusion with simulation CT images were performed to assist the targets delineation.

All patients were treated with IMRT definitively. The details of the tumor volume delineation have been detailed previously [14]. The doses prescribed to PTV_GTV-P_, PTV_GTV-N_ and PTV_CTV-H_ was 70 Gy, 68.25/66.50 Gy, and 61.25 Gy (in 35 fractions). The low neck or supraclavicular field (PTV_CTV-L_) was treated with AP/PA fields and received 30 fractions of 1.8 Gy/fraction, for a total of 54 Gy. Radiotherapy was delivered once daily, 5 fractions per week, over 7 weeks.

**Figure 1:**
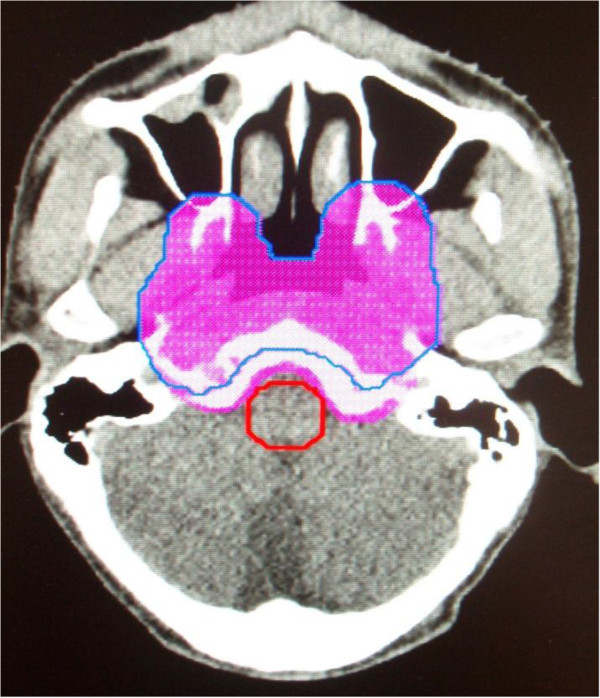
**An example of the contour of the PTV**_**GTV-P**_**.** The aubergine shadow represented the PTV_GTV-P1_ and the blue line was PTV_GTV-P2_. The margin between tumor and critical normal was extended.

**Figure 2:**
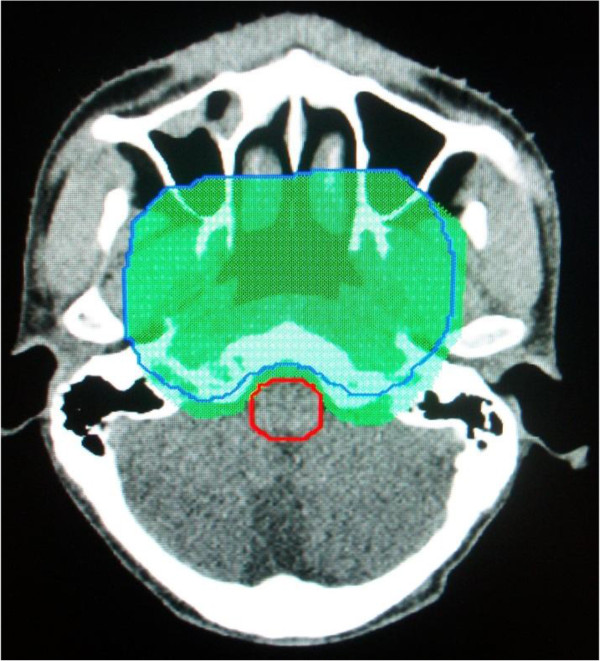
**An example of the contour of the PTV**_**CTV-H**_**.** The green shadow represented the PTV_CTV-H1_ and the blue line was PTV_CTV-H2_. The margin between tumor and critical normal was extended.

When there was a safe margin between the target and the critical normal tissues (e.g. brain stem), the outlined of the GTV (GTV-P1) and CTV-H (CTV-H1) were mainly based on the enhanced MRI before the neoadjuvant chemotherapy; otherwise, the enhanced MRI after the neoadjuvant chemotherapy. The doses prescribed to PTV_GTV-P1_, and PTV_CTV-H1_ was 56 Gy and 49 Gy (in 28 fractions). Inverse planning was used. Before the 23th fraction of IMRT, a new nasopharyngeal enhanced MRI acquired for all patients. GTV-P and CTV-H were modified based on the tumor shrinkage shown on the new nasopharyngeal enhanced MRI and reoutlined on the original CT simulation scan. Replanning was performed and the doses prescribed to new PTV_GTV-P2_ and PTV_CTV-H2_ was 14 Gy and 12.25 Gy (in 7 fractions). GTV-LN and CTV-L were not changed. All plans were created on the Pinnacle. For all plans, 7–9 coplanar 6-MV photon beams were evenly distributed around the patient’s head and neck. Examples of contoured PTV_GTV-P2_ and PTV_CTV-H2_ were presented in Figures 1 and 2.

### Volume and dosimetric comparisons

Target volumes (PTV_GTV-P1_ vs. PTV_GTV-P2_; PTV_CTV-H1_ vs. PTV_CTV-H2_) were compared with a paired samples analysis. For each IMRT plan, dose–volume histograms (DVHs) were calculated for target volumes and normal structures. DVHs of target volumes and all sensitive structures were characterized by multiple endpoints. The endpoints of PTV_GTV-P_ and PTV_CTV-H_ were the mean dose and the doses encompassing 95% (D-95) and 99% (D-99) of the volumes. To reflect the characteristics of DVHs in high dose regions for serial structures including the brainstem, optic chiasm and optic nerves, we chose three endpoints that were the maximum dose, the doses encompassing 5% and 10% of the volumes. For sensitive structures with functional subunit in parallel such as the temporal lobe, inner and middle ear, the mean dose and the doses encompassing 50% and 80% of the volumes were chosen as endpoints. The DVHs of IMRT plan with replanning were compared to the IMRT plan without replanning by analysis of paired samples *t* test. A value of p < 0.05 was considered statistically significant.

### Statistical analysis

The Statistical Package for the Social Science (SPSS for Windows 16.0; SPSS Inc., Chicago, IL) was used for statistical analysis. Descriptive statistics were used to summarize the patient characteristics. The estimated overall survival (OS), disease progression-free survival (PFS), local progression-free survival (LPFS), regional progression-free survival (RPFS), and distant metastasis-free survival (DMFS) were calculated by the Kaplan-Meier method. Adverse events were evaluated according to the National Cancer Institute’s Common Terminology Criteria for Adverse Events (NCI CTCAE 3.0). The duration of survival was measured from the time of pathological diagnosis until death or the date of the last follow-up visit for patients still alive.

### Follow-up

After the completion of concurrent chemoradiation, all patients were assessed every 3 months during the first 3 years, every 6 months for the next 2 years, and annually thereafter. All local recurrences were diagnosed with nasopharyngoscopy and biopsy, MRI of the head and neck. Regional recurrences were diagnosed by clinical examination of the neck and, in doubtful cases, by fine needle aspiration or an MRI scan of the neck. Distant metastases were diagnosed by clinical symptoms, physical examinations, and imaging methods. Whenever necessary and possible, salvage treatments including re-irradiation, chemotherapy, and surgery, was provided to patients with relapse.

## Results

### Patient characteristics

From June 2007 to January 2012, 32 patients (median, 48 years; range, 20-66 years) with intracranial invasion NPC treated with replanning IMRT were analyzed for this research. All patients were treated with neoadjuvant chemotherapy and concurrent chemoradiotherapy. The median follow-up was 19.5 months (range 8.0–63.0). Of the 32 patients, 24 were male and 8 were female (male:female ratio, 3:1) and 7 were in N0, 9 were in N1, 12 were in N2, and 4 were in N3.

## Dose–volume details

### Volumetric changes and dosimetry comparisons of target volumes

The mean volume of PTV_GTV-P2_ (116.09 ± 37.69 cc) decreased significantly (p = 0.000), compared with PTV_GTV-P1_ (102.92 ± 30.64 cc). The mean volume to the PTV_CTV-H1_ and PTV_CTV-H2_ were 509.36 ± 143.09 cc and 486.62 ± 136.84 cc (p = 0.000), respectively.

For both the PTV_GTV-P2_ and the PTV_CTV-H2_, the mean dose, D-95, D-99, and V95 (percent of volume receiving ≥95% of the prescribed dose) were not different between replanning and not replanning (Table 1). The differences were significant for both the PTV_GTV-P1_ and the PTV_CTV-H1_ as we expected (Table 1).

**Table 1:** Dosimetry comparisons of target volumes

**Variable**	**Replanned**	**Not replanned**	***P *** **value**	**Variable**	**Replanned**	**Not replanned**	***P *** **value**
**PTV**_**GTV-P1**_				**PTV**_**GTV-P2**_			
D-mean (Gy)	71.86 ± 0.88	72.05 ± 0.98	0.000	D-mean (Gy)	72.29 ± 1.11	72.32 ± 1.07	0.394
D-95 (Gy)	67.15 ± 2.30	68.17 ± 1.68	0.000	D-95 (Gy)	69.41 ± 1.38	69.28 ± 1.19	0.276
D-99 (Gy)	63.52 ± 3.59	65.64 ± 2.08	0.000	D-99 (Gy)	67.29 ± 1.62	67.16 ± 1.50	0.463
V95 (%)	96.29 ± 3.07	97.42 ± 2.50	0.000	V95 (%)	99.20 ± 1.52	99.66 ± 2.47	0.208
PTV_CTV-H1_				PTV_CTV-H2_			
D-mean (Gy)	66.54 ± 1.37	66.70 ± 1.40	0.363	D-mean (Gy)	66.65 ± 1.17	66.82 ± 1.40	0.184
D-95 (Gy)	60.35 ± 1.35	61.26 ± 1.80	0.015	D-95 (Gy)	61.15 ± 1.18	61.43 ± 1.79	0.435
D-99 (Gy)	55.36 ± 3.17	58.20 ± 2.82	0.000	D-99 (Gy)	58.10 ± 2.65	58.54 ± 2.76	0.284
V95 (%)	97.69 ± 1.53	98.89 ± 0.78	0.000	V95 (%)	98.95 ± 1.29	99.13 ± 0.73	0.423

### Dosimetry comparisons of normal tissues

#### Serial structures

Among the serial-sensitive structures evaluated, replanning reduced the dose of brain stem, optic nerve and optic chiasm (Table 2). There was a significant decrease with the replanning IMRT plan in the dose delivered to the brain stem when analyzed by doses encompassing 5% of the volume (p = 0.001) and 10% of the volume (p = 0.023).

**Table 2:** Dosimetry comparisons of serial structures and parallel structures

**Variable**	**Replanned**	**Not replanned**	**P value**	**Variable**	**Replanned**	**Not replanned**	**P value**
Brain stem				Temporal lobe			
D1 (Gy)	56.45 ± 3.33	57.31 ± 5.65	0.209	D-Mean (Gy)	20.20 ± 3.26	21.48 ± 3.86	0.000
D5 (Gy)	52.76 ± 3.28	54.09 ± 4.34	0.001	D50 (Gy)	14.87 ± 4.75	16.05 ± 5.23	0.000
D10 (Gy)	50.38 ± 3.36	51.40 ± 4.36	0.023	D80 (Gy)	6.53 ± 2.66	6.96 ± 2.88	0.000
Optic chiasm				Inner ear			
D1 (Gy)	58.55 ± 7.79	62.76 ± 7.64	0.000	D-Mean (Gy)	52.96 ± 4.94	53.41 ± 5.23	0.018
D5 (Gy)	57.86 ± 7.63	62.00 ± 7.47	0.000	D-50 (Gy)	52.83 ± 5.34	53.98 ± 5.81	0.009
D10 (Gy)	57.41 ± 7.66	61.30 ± 7.58	0.000	D-80 (Gy)	45.82 ± 5.43	46.28 ± 5.73	0.030
Optic nerve				Middle ear			
D1 (Gy)	57.77 ± 8.95	61.74 ± 8.45	0.000	D-Mean (Gy)	59.24 ± 5.53	59.51 ± 5.76	0.097
D5 (Gy)	56.31 ± 8.96	60.29 ± 8.49	0.000	D-50 (Gy)	59.52 ± 5.81	60.00 ± 6.09	0.009
D10 (Gy)	54.83 ± 9.15	58.75 ± 8.72	0.000	D-80 (Gy)	54.39 ± 6.71	54.79 ± 6.97	0.027

*Abbreviations*: *D1* dose to 1% of the volume, *D5* dose to 5% of the volume, *D10* dose to 10% of the volume, *D50* dose to 50% of the volume, *D80* dose to 80% of the volume, *Dmean* mean dose per fraction.

The D-1, D-5 and D-10 of the brain stem decreased with replanning. There were significantly different between the replanning and without replanning in the endpoint doses (the maximum dose, the doses encompassing 5% and 10% of the volume) to the chiasm and the optic nerve for each of the endpoints considered (all p = 0.000).

#### Parallel structures

In comparison between the replanning and without replanning IMRT plans for parallel sensitive structures, there were statistical differences to temporal lobe for each of the endpoints considered (the maximum dose, the doses encompassing 50% and 80% of the volume), as well as the middle ear and inner ear. The details of the comparison of average endpoint doses for selected parallel structure were shown in Table 2.

## Treatment outcomes

### Response of disease

Objective responses were 93.7% (CR 15.6%) and 100% (CR 12%) to the primary tumor and cervical lymph nodes after neoadjuvant chemotherapy, the corresponding rates were 100.0% (CR 100%) and 100% (CR 100%) 3 months after completion of radiotherapy.

### Acute toxicity

Overall, the regimen was tolerated. All patients completed at least 2 courses neoadjuvant chemotherapy, and 30 (93.7%) patients completed 3 planned courses. Chemotherapy dosage was decreased in 17 (53.1%) patients due to severe adverse events, including 9 patients in the second course and 8 in the third course.

The most commonly severe (grade 3–4) haematological and nonhaematological adverse events were neutropenia (24 patients; 75%) and Xerostomia (9 patients; 28.1%). Grade 3–4 adverse events of neoadjuvant chemotherapy were listed in Table 3. No grade 5 toxicity occurred.

**Table 3:** Grade 3–4 treatment-related acute adverse events

	**During neoadjuvant chemotherapy**		**During concurrent chemoradiation**	
**Event**	**Grade 3**	**Grade 4**	**Grade 3**	**Grade 4**
Hematological				
Leukopenia	15(46.8%)	3(9.3%)	7(21.8%)	1(3.1%)
Neutropenia	6(18.7%)	18(56.2%)	2(6.2%)	4(12.5%)
Neutropenia fever	3(9.3%)	0(0.0%)	0(0.0%)	0(0.0%)
Thrombocytopenia	0(0.0%)	0(0.0%)	3(9.3%)	1(3.1%)
Anemia	0(0.0%)	0(0.0%)	2(6.2%)	0(0.0%)
Non-hematological				
Fatigue	4(12.5%)	0(0.0%)	4(12.5%)	0(0.0%)
Nausea/vomiting	4(12.5%)	0(0.0%)	4(12.5%)	0(0.0%)
Ototoxicity	0(0.0%)	0(0.0%)	0(0.0%)	0(0.0%)
Diarrhea	0(0.0%)	0(0.0%)	0(0.0%)	0(0.0%)
Liver dysfunction	0(0.0%)	0(0.0%)	0(0.0%)	0(0.0%)
Kidney dysfunction	0(0.0%)	0(0.0%)	0(0.0%)	0(0.0%)
Stomatitis	1(3.1%)	0(0.0%)	8(25%)	0(0.0%)
Dermatitis	-	-	1(3.1%)	0(0.0%)
Xerostomia	-	-	9(28.1%)	0(0.0%)

The median course of concurrent chemotherapy was 4 (range 2-6). The number of patients who finished at least 5 or 4 courses of concurrent chemotherapy totaled 16 (50.0%) and 21 (65.6%), respectively. All patients experienced some degree of acute toxicity during concurrent chemoradiotherapy (Table 3).

### Survival rates

**Figure 3:**
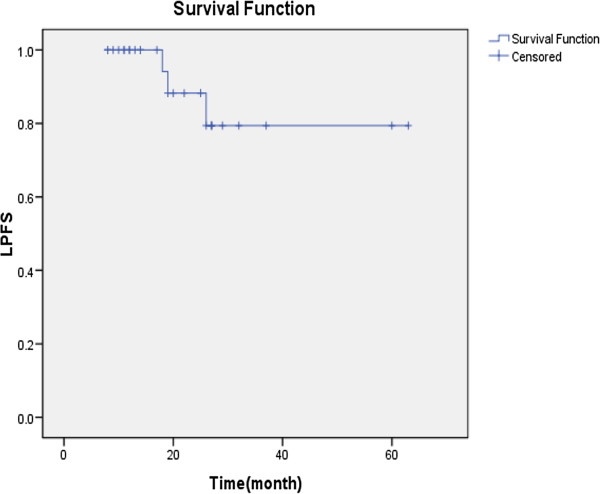
Kaplan–Meier estimators of local progression-free survival (LPFS) for NPC patients.

A total of 5 failures were observed during follow up, including 3 patients with local recurrence alone and 2 patients with distant metastasis alone. The 2-year PFS was 77.7% (95%CI, 57.6% to 96.4%), LPFS 88.2% (95%CI, 72.9% to 100.0%), RPFS 100.0%, DMFS 89.6% (95%CI, 75.9% to 100.0%) and OS 100.0%, respectively. Figure 3 shows the Kaplan-Meier curves for LPFS.

## Discussion

Patients with extensive local disease infiltrating or abutting the critical tissues are one of the most difficult groups to treat. To maximize their chance of cure and spare the critical normal, we used neoadjuvant chemotherapy in an attempt to shrink the primary tumor and replanning during concurrent chemotherapy with IMRT technique for wider margin. The primary objective of this study we are reporting addressed the potential dosimetry benefit to critical normal tissue of neoadjuvant chemotherapy and replanning IMRT. The second objective addressed the efficacy of this treatment strategy. Our results suggest that neoadjuvant chemotherapy and replanning IMRT help to ensure safe does to normal structures for NPC with intracranial invasion. The doses to many critical normal structures were significantly decreased. Two-year clinical outcomes were encouraging.

Radiation therapy in concurrent with cisplatin based chemotherapy is the standard treatment of choice for T3/4 and/or N + NPC [15-17]. The role of neoadjuvant chemotherapy is controversy. The results of phase III trials and meta-analysis have demonstrated that neoadjuvant chemotherapy decreased locoregional failures as well as metastases, without benefit on OS for locoregional advanced NPC. However, none of the trials used concurrent chemotherapy. The reasons that neoadjuvant therapy has failed to demonstrate OS efficacy may possibly be due to inadequate neoadjuvant chemotherapy intensity or efficacy and inadequacy of the local therapies. So, combining more effective neoadjuvant chemotherapy with concurrent chemoradiotherapy is hypothesized to be a promising strategy. New combinations, such as TPF regimene (docetaxel or paclitaxel, platinum, and 5-FU) have been shown to be superior to standard PF regimen (platinum, and 5-FU) for squamous cell carcinoma of the head and neck (HNSCC) in numerous phase III trials. And several studies [11,12,18] on neoadjuvant setting involving taxane provided impressive results in NPC patients. A randomized phase II trial [12] published in 2009 studied the stage III ~ IVB NPC patients treated with or without neoadjuvant chemotherapy using docetaxel and cisplatin (TP regimen) followed by concurrent cisplatin chemotherapy and radiation. The researchers demonstrated a significant improvement in overall survival in patients treated with neoadjuvant TP chemotherapy: The 3-year OS rate of 94.1% after neoadjuvant chemotherapy followed by chemoradiation, as compared to 65% after chemoradiation alone. In 2007, 2 prospective phase II clinical trials respectively for stage III and non-metastatic stage IV NPC were initiated to evaluate the efficacy and safety of induction TPF chemotherapy followed by concurrent CRT using 3D-CRT or IMRT in our hospital. The overall response rate in the primary site and neck region were 97.4% (CR 24.1%) and 100% (CR 12.1%) after completion of neoadjuvant chemotherapy. With median follow-up times of 32.9 months, the 3-year overall survival rates were 94.8% (95% CI 87.6%-100%) and 90.2% (95% CI 81.8%-98.6%) for stage III and IVA/B NPC patients, respectively. Sixty-four patients in this reporting group were included in the prospective phase II clinical trial for stage IV NPC. The overall response rate and the OS of this retrospective study were similar with that of our prospective studies and Hui’s [12] study.

It was reported in several previous studies that many patients undergoing RT for HNSCC or NPC had significant anatomic changes during their course of treatment, including shrinking of the primary tumor or nodal masses and in overall body weight loss, especially for patients with large lymph node in the neck [9,10,19]. Thus, repeat CT imaging and IMRT replanning during the course of IMRT for selected patients are essential to identify dosimetric changes and to ensure adequate doses to target volumes and safe doses to normal tissues and recommendatory for specific patients [4,20]. However, none of these reports have studied anatomic and dosimetric changes in patients receiving neoadjuvant chemotherapy. Anatomic changes in the external contour and shape are the main reasons for the repeat CT. And shrinkage of the primary tumor or nodal masses is the main reason for anatomic changes. The objective responses were 93.7% (CR 15.6%) and 100% (CR 12%) to the primary tumor and cervical lymph nodes after neoadjuvant chemotherapy in this reporting group patients, namely almost all patients developed significant tumor shrinkage before IMRT. On the one hand, the shrinkage of the tumor facilitated the IMRT planning; on the other hand, shrinkage of the primary tumor or nodal masses and anatomic changes in the external contour and shape during the latter IMRT might be minimal, which makes repeat CT imaging during radiotherapy not as necessary as we expected in previous reports. Moreover, the implementation of repeat CT imaging increased physician time spent in recontouring normal structures and cost to patients. As a result, we did replanning IMRT without repeat CT imaging for our patients.

Our dosimetric results showed that IMRT replanning was beneficial to ensure safe doses to normal structures for patients with intracranial invasion NPC. All IMRT plans provided good coverage of the target volumes. With replanning during IMRT, the doses to critical normal structures (brain stem, optic chiasm, optic nerve, inner ear, middle ear) were significantly decreased (Table 2). Decreased doses to critical normal structures may help to alleviate late toxicities. Due to the relatively short follow-up time in this reporting, the late toxicities need longer follow-up and will be the important clinical outcomes in our update report.

One important issue arising from our study is how to delineate tumor volumes. It is unknown whether or not it is safe (in terms of local-regional control) to contour GTV based on MRI scans after the neoadjuvant chemotherapy and decrease the size of the GTV during the course of fractionated radiotherapy for NPC. For our group patients, initial GTV not only included all visible tumors and/or enlarged regional lymph nodes as determined by contrast-enhanced MRI after the neoadjuvant chemotherapy, but also the invaded skull base showed on MRI before neoadjuvant chemotherapy with caution. Hansen et al. [20] chose to maintain the size of the original GTV when contouring the GTV on the anatomy of the second CT scans. Zhao et al. [21] chose to adapt the GTV to the observed tumors or lymph nodes volumes on the anatomy of the second CT scans, but maintain the size of the original CTV. We intentionally chose to maintain the size of the GTV and CTV as possible as we can when contouring the GTV-P2 and CTV-H2 although the tumor shrunk during radiotherapy. We reduce the target volumes unless there is no safe margin between the target and the critical normal tissues (Figures 1 and 2). As far as T4 NPC is concerned, the 2-year LRFS (88.2%) in our group is excellent, and 3 patients had local recurrence in the GTV-P2, which suggested that this target contouring was feasible, but need further confirmed.

In conclusion, neoadjuvant chemotherapy and replanning IMRT according to tumor shrinkage during the course of IMRT is essential to ensure adequate doses to safe doses to normal tissues for intracranial invasion NPC. This treatment strategy is well tolerated and produces encouraging outcome. However, it remains to be proven in the long-term outcomes and late complications.

## Competing interests

The authors declare that they have no competing interests.

## Authors’ contributions

XN carried out the study designing and data collecting, participated in the data analysing and drafted the manuscript. XC participated in the research implementation. YG participated in the research implementation. CH carried out the study designing. LK carried out the study designing and data collecting, participated in the data analysing and revised the manuscript. All authors read and approved the final manuscript.

## References

[B1] Ng WT Lee MC Hung WM Clinical outcomes and patterns of failure after intensity-modulated radiotherapy for nasopharyngeal carcinoma Int J Radiat Oncol Biol Phys 2011 79 420 428 10.1016/j.ijrobp.2009.11.024 20452132

[B2] Kam MK Chau RM Suen J Choi PH Teo PM Intensity-modulated radiotherapy in nasopharyngeal carcinoma: dosimetric advantage over conventional plans and feasibility of dose escalation Int J Radiat Oncol Biol Phys 2003 56 145 157 10.1016/S0360-3016(03)00075-0 12694833

[B3] Xia P Fu KK Wong GW Akazawa C Verhey LJ Comparison of treatment plans involving intensity-modulated radiotherapy for nasopharyngeal carcinoma Int J Radiat Oncol Biol Phys 2000 48 329 337 10974445 10.1016/s0360-3016(00)00585-x

[B4] Wang W Yang H Hu W Clinical study of the necessity of replanning before the 25th fraction during the course of intensity-modulated radiotherapy for patients with nasopharyngeal carcinoma Int J Radiat Oncol Biol Phys 2010 77 617 621 10.1016/j.ijrobp.2009.08.036 20138444

[B5] Lee AW Lau KY Hung WM Potential improvement of tumor control probability by induction chemotherapy for advanced nasopharyngeal carcinoma Radiother Oncol 2008 87 204 210 10.1016/j.radonc.2008.02.003 18329742

[B6] Chau RM Leung SF Kam MK A broadly adaptive array of dose-constraint templates for planning of intensity-modulated radiation therapy for advanced T-stage nasopharyngeal carcinoma Int J Radiat Oncol Biol Phys 2009 74 21 28 10.1016/j.ijrobp.2008.07.041 19171440

[B7] Wolden SL Chen WC Pfister DG Kraus DH Berry SL Zelefsky MJ Intensity-modulated radiation therapy (IMRT) for nasopharynx cancer: update of the Memorial Sloan-Kettering experience Int J Radiat Oncol Biol Phys 2006 64 57 62 10.1016/j.ijrobp.2005.03.057 15936155

[B8] Kam MK Teo PM Chau RM Treatment of nasopharyngeal carcinoma with intensity-modulated radiotherapy: the Hong Kong experience Int J Radiat Oncol Biol Phys 2004 60 1440 1450 10.1016/j.ijrobp.2004.05.022 15590175

[B9] Bhide SA Davies M Burke K Weekly volume and dosimetric changes during chemoradiotherapy with intensity-modulated radiation therapy for head and neck cancer: a prospective observational study Int J Radiat Oncol Biol Phys 2010 76 1360 1368 10.1016/j.ijrobp.2009.04.005 20338474

[B10] Wu Q Chi Y Chen PY Krauss DJ Yan D Martinez A Adaptive replanning strategies accounting for shrinkage in head and neck IMRT Int J Radiat Oncol Biol Phys 2009 75 924 932 10.1016/j.ijrobp.2009.04.047 19801104

[B11] Johnson FM Garden AS Palmer JL A phase I/II study of neoadjuvant chemotherapy followed by radiation with boost chemotherapy for advanced T-stage nasopharyngeal carcinoma Int J Radiat Oncol Biol Phys 2005 63 3 717 724 10.1016/j.ijrobp.2005.03.001 16199307

[B12] Hui EP Ma BB Leung SF Randomized phase II trial of concurrent cisplatin-radiotherapy with or without neoadjuvant docetaxel and cisplatin in advanced nasopharyngeal carcinoma J Clin Oncol 2009 27 242 249 10.1200/JCO.2008.18.1545 19064973

[B13] Fang FM Tsai WL Go SF Implications of quantitative tumor and nodal regression rates for nasopharyngeal carcinomas after 45 Gy of radiotherapy Int J Radiat Oncol Biol Phys 2001 50 961 969 10.1016/S0360-3016(01)01531-0 11429224

[B14] Kong L Zhang YW Hu CS Neoadjuvant chemotherapy followed by concurrent chemoradiation for locally advanced nasopharyngeal carcinoma Chin J Cancer 2010 29 5 551 555 10.5732/cjc.009.10518 20426907

[B15] Lin J-C Adjuvant chemotherapy in advanced nasopharyngeal carcinoma based on plasma EBV load J Radiat Oncol 2012 1 117 127 10.1007/s13566-012-0036-9

[B16] Lee AWM Ng WT Chan OSH If concurrent–adjuvant chemoradiotherapy is beneficial for locoregionally advanced nasopharyngeal carcinoma, would changing the sequence to induction–concurrent achieve better outcome? J Radiat Oncol 2012 1 107 115 10.1007/s13566-012-0032-0

[B17] Wang TJC Riaz N Cheng SK Intensity-modulated radiation therapy for nasopharyngeal carcinoma: a review J Radiat Oncol 2012 1 129 146 10.1007/s13566-012-0020-4

[B18] Chan AT Ma BB Lo YM Phase II study of neoadjuvant carboplatin and paclitaxel followed by radiotherapy and concurrent cisplatin in patients with locoregionally advanced nasopharyngeal carcinoma: therapeutic monitoring with plasma Epstein-Barr virus DNA J Clin Oncol 2004 22 15 3053 3060 10.1200/JCO.2004.05.178 15284255

[B19] Barker JJ Garden AS Ang KK Quantification of volumetric and geometric changes occurring during fractionated radiotherapy for head-and-neck cancer using an integrated CT/linear accelerator system Int J Radiat Oncol Biol Phys 2004 59 960 970 10.1016/j.ijrobp.2003.12.024 15234029

[B20] Hansen EK Bucci MK Quivey JM Repeat CT imaging and replanning during the course of IMRT for head-and-neck cancer Int J Radiat Oncol Biol Phys 2006 64 355 362 10.1016/j.ijrobp.2005.07.957 16256277

[B21] Zhao L Wan Q Zhou Y The role of replanning in fractionated intensity modulated radiotherapy for nasopharyngeal carcinoma Radiother Oncol 2011 98 23 27 10.1016/j.radonc.2010.10.009 21040992

